# Supplementary material for: Childhood brain morphometry in children with persistent stunting and catch-up growth
Source: PLoS One. 2025 Feb 11;20(2):e0306500. doi: 10.1371/journal.pone.0306500 (PMC11813088; doi:10.1371/journal.pone.0306500)
Supplement: Attachment — Submitted filename: Response_to_Reviewers.docx [file pone.0306500.s001.docx]

To,

The Editor, Reviewers,

PLOS ONE

Date: 23 August 2024

**Subject: Addressing Editor/Reviewer Comments for PONE-D-24-24149**

Dear Editor, Reviewers,

Many thanks to you for your helpful feedback. We have addressed all your concerns and changes are highlighted in the manuscript. Responses to concerns are highlighted in red in this document. Let us know if you have any further queries.

Thank you.

Kind regards,

Authors of PONE-D-24-24149

1)In Abstract- Please specify the methodology as suggested by reviewer 4

Answer: We have added the methodology used in the study, under the “Methods” section in the “Abstract”.

Also In Introduction- Why Freesurfer was used to study morphometry..add your motivation?

Answer: We have added our motivation for using Freesurfer in this study in the MRI Image Acquisition and Processing paragraph under the “Materials and Methods” section. Freesurfer has been chosen for this study due to its widespread acceptance as a standard for brain volumetric studies and its validation for use in children aged 4 to 11 years. (258-260)

2)Why Desikan killiany atlas was used...Any reason or reference?

Answer: We have added the reason for using Desikan Killiany atlas in the MRI Image Acquisition and Processing paragraph under the “Materials and Methods” section. The selection of the Desikan-Killiany atlas for this study was based on its consistent representation of atrophy levels and accurate depiction of the macroscopic anatomy of gyri and sulci. This made it a preferred choice over other atlases for examining both healthy children and those with stunted growth. (266-269)

3)Studies have shown sex and age differences as well as sex by age interaction for children.. See below references

https://pubmed.ncbi.nlm.nih.gov/23500670/

https://doi.org/10.1093/cercor/11.6.552

I am not sure if sex by age interaction was considered in your model?

Answer: Sex by age has not been considered in our analyses as most of the scans were done at the Age of 9 and only a few were done after a year, due to disruption caused by Covid-19. Therefore, we had adjusted for sex and age separately in our model.

4)You seem to have missed some latest studies. If relevant discuss these in introduction or discussion sections as appropriate in context of their results.

https://www.sciencedirect.com/science/article/pii/S0361923023002721

https://www.ncbi.nlm.nih.gov/pmc/articles/PMC10964341/

Answer: Thank you for suggesting the above studies. The above studies have focused on studying brain morphometry related to Chronic Hepatic Encephalopathy and Pediatric Hydrocephalus, while the current study lays emphasis on studying the effect of early childhood stunting on brain morphometry. In relation to that, we have reviewed literatures that had focused on outcomes related to childhood stunting.

Reviewer #1: This work studies early childhood stunting effects. The authors highlighted that this is the first neuroimaging analysis to investigate the effects of childhood stunting. Specifically, this study analyses correlations between childhood stunting occurrence and multiple covariants, including total brain volume, subcortical volume, bilateral cerebellar white matter, posterior corpus callosum, etc. Statistical analyses including p-value, are conducted, categorized by different regions.

I think this work is coherent and supportive and can provide useful references for future research on the reasonings and trends of stunting effects.

Answer: Thank you for your encouraging comments.

Reviewer #2: This study provides valuable insights into the long-term neurological impacts of early childhood stunting and subsequent catch-up growth, an area with limited prior data.

While this study offers important insights, there are several limitations to consider. The sample size of 178 children, though informative, may not fully capture the variability within the broader population.

Additionally, the study is geographically limited to Vellore, south India, which might affect the generalizability of the findings to other regions or populations.

Answer: Thank you. We agree with limitations suggested and have included these in limitations in the study (lines 540-41).

The cross-sectional nature of the neuroimaging analysis at nine years of age does not allow for the assessment of brain development trajectories over time.

Answer: Thank you. We agree. In the same birth cohort, we have recently completed 12-year neuroimaging analysis, which will be further evaluated for a possible trajectory evaluation.

Furthermore, potential confounding factors such as socioeconomic status, educational environment, and genetic predispositions were not thoroughly addressed, which could influence the observed outcomes.

Answer: Thank you. We agree with the observation. We have another publication from the same cohort “Koshy B, Srinivasan M, Gopalakrishnan S, Mohan VR, Scharf R, Murray-Kolb L, et al. Are early childhood stunting and catch-up growth associated with school age cognition?-Evidence from an Indian birth cohort. PloS one. 2022;17(3):e0264010. Epub 20220302. doi: 10.1371/journal.pone.0264010. PubMed PMID: 35235588; PubMed Central PMCID: PMCPMC8890627.”, which has evaluated these factors. Persistent stunting had an effect on both verbal and performance quotient by around 5 points even after correction with SES and maternal cognition. The current paper is a follow-up analysis of the previous published analysis.

Most of the references are clubbed. Its better to use one citation at the end of one line.

Answer: Thank you. We have tried to give specific references now.

Reviewer #3: 1.1. Current literature has established that handedness is associated with differences in brain morphology. Given that the current study has only included one left-handed participant, rather than including that participant, it may be worth removing that participant.

Answer: Thank you for your comment. We agree that handedness play a role in differences in brain morphology based on current literature. But from our field experience, we have noted that many study children, who were born left-handed, had been trained to be right-handed by their parents. Therefore, we chose not to add handedness as a confounding factor between the association of early childhood stunting and brain morphometry, and also keep the corresponding participant in the analysis.

1.2. From the literature review/introduction, I understand that there are very few work looking at childhood stunting and brain morphometry. However, it would have been beneficial to include a broader range of knowledge, such as studies examining malnutrition (which is closely related to stunting) and brain morphometry, or research on childhood stunting and its effects on other brain modalities, such as fMRI.

Answer: Thank you. We agree with the reviewer. We have referenced work done in preterm babies as this is the closest set available for comparison.

1.3. The study mentions addressing the research gap of limited data on the effects of early childhood stunting and catch-up growth on brain morphometry. However, the conclusion primarily emphasized the differences between the 'Never Stunted' and 'Always Stunted' children. The 'caught-up' groups were not discussed in detail, and there was limited interpretation of the findings related to these groups.

Answer: Our analysis revealed a decrease in the volumes of different brain regions across the groups, but we found statistically significant differences only between the 'Never Stunted' and 'Always Stunted' groups. However, we had explored the dose-response exhibited by brain regions due to catch-up growth and discussed the role of nutritional factors in catch-up growth. We acknowledge that in the "Discussion" section, we missed to include the catch-up growth groups in some comparisons, which has now been addressed. (474-476; 524-525)

2.1. Aside from age and gender, total intracranial volume is also an important covariate for volumetric analyses, which I don't think has been considered in the current study. In addition, I am curious whether the present study has considered the child's awake/asleep state during the MRI scan as a covariate.

Answer: Thank you. All children included in the study were asleep during MRI scanning. The current cohort is a low-and-middle-income cohort with a high percentage of microcephaly, mostly guided by parental head circumference – this is already published (Sindhu KN, Ramamurthy P, Ramanujam K, Henry A, Bondu JD, John SM, **Koshy B**, Bose A, Kang G and Mohan VR. Low head circumference during early childhood and its predictors in a semi-urban settlement of Vellore, Southern India. BMC Pediatrics2019.19:182. https://doi.org/10.1186/s12887-019-1553-0)

2.2. I understand that visual checks have been carried out visually, but I am wondering if post segmentation checks have been performed (e.g., using Qoala-T on the Freesurfer output) as the study has not mentioned and it will be important information.

Answer: Thank you for your suggestion. We have used Qoala-T on the freesurfer output for assessing the accuracy of visual quality checks of the scans. The process and the results have been described in the “MRI Image Acquisition and Processing” sub-section and in the “Results” section. (262-265)

3. Authors have stated that some restrictions will apply.

Answer: Thank you. We function within the Government of India regulations and are happy to share datasets in line with Indian Council of Medical Research guidelines.

4. There are some punctuation and grammatical errors, which may be a result of the long sentences. For example - "In a previous report from the same cohort, children who remained persistently stunted had a significant reduction in verbal, and total cognition scores by 4·6 points which corresponded to a 0·3 standard deviation difference compared to those never stunted, and is consistent with the previous literature [6, 17]." The comma is misplaced, and there are grammatical errors: "In a previous report from the same cohort, children who remained persistently stunted had significant reductions in verbal and total cognition scores, by 4·6 points which corresponded to a 0·3 standard deviation difference compared to those children who have never stunted, and are consistent with the previous literature [6, 17]."

Answer: The corrections have been made in the manuscript. (458-461)

5. I was curious on why the study looked till 9 years old, and not an older age since the study was interested in the catch-up growth on brain morphometry (for example, 12 years old is an age at which brain volume is suggested to continue growing till).

Answer: Thank you for your feedback. We are already being following up the participants till 12 years old and looking at their brain volumes, in lieu of their performances in psychological assessments.

Reviewer #4: The work by Koshy et al to understand the morphometric changes associated with stunting and catch-up growth is interesting with a larger Indian cohort from a small town. Though I liked the approach but the study needs considerable improvement from the analysis point of view.

MAJOR

Abstract- Please specify the methodology used in the method section to let readers understand what was done before they understand the finding. Please specify multiple comparison etc here

Answer: We have added the methodology used in the study, under the “Methods” section in the “Abstract”. (152-154)

Introduction-

1. Please define stunting and the criteria to identify

2. The statement 1000days = 2 years is bit confusing.

Answer: We have added the explanation for stunting and have corrected the statement regarding 1000 days = 2 years in the “Introduction” section. (178-180)

Method

3. Every study should be an independent read. So please briefly mention the cohort details in main manuscript or in supplement.

Answer: We have added the cohort details in the main manuscript under the “Materials and Methods” section and the “Results” section. (226-232; 304-310)

4. Please mention the age at which the childrens were scanned. Additionally, provide details on How many children needed Triclofos- Please mention.

Answer: We have added the age details in the “MRI Image Acquisition and Processing” section and also the number of children needing Triclofos in the “Results” section. (319)

5. Are the Regional Brain volumes normalised. Most of regional level results will dissapear if Total brain volume is regressed.

Answer: Thank you. The current cohort is a low-and-middle-income cohort with a high percentage of microcephaly, mostly guided by parental head circumference – this is already published (Sindhu KN, Ramamurthy P, Ramanujam K, Henry A, Bondu JD, John SM, **Koshy B**, Bose A, Kang G and Mohan VR. Low head circumference during early childhood and its predictors in a semi-urban settlement of Vellore, Southern India. BMC Pediatrics2019.19:182. https://doi.org/10.1186/s12887-019-1553-0).

6. How was the Time of MRI aquisition adjusted.

Answer: We have not adjusted for Time of MRI acquisition in this study as the same protocol has been followed for all participants without any deviation. Similarly, there was no time differences reported after MRI acquisition for all participants.

Result

Please provide all the details of ANNOVA analysis for each group. Please adjust the time of aquistion, TIV and results will get shifted from present. This will provide a robust picture of the analysis.

Answer: The ANOVA analyses were conducted separately to compare each stunting group with the healthy group. The p-value was determined for each group to demonstrate the decrease in brain volumes. Age and sex were adjusted as co-variates for each analysis. We have also explained these details in the "Materials and Methods" section, under the "Statistical Analyses" subsection. In the current study, we opted to focus on comparing individual stunting groups with the healthy group, subsequently only including the p-value rather than the DF, SS, and F-values. While including these would have provided a more comprehensive analysis had we assessed all groups collectively, for our specific investigation, individual group analyses were deemed more suitable for the looking into the impact of catch-up growth.

MINOR

(1) Writing needs to be improved through out the manuscript at several places- starting from abstract to discussion- For example- Amongst 251 children from the overall cohort, 178 children with a mean age of 9.54 were considered for further analysis.- It sounds like the volumetric parameter is assessed longitudinally.

Please write the message in each Paragraphs clearly and directly. For example Paragraph 3 shows Neuroimaging is expensive which I agree, but authors themselves have used neuroimaging rather than any alternative.

Answer: We have made the recommended changes to the writing. (156-157; 207)
